# Risk Factors of a Serious Underlying Cause of Palpitations Among Patients Attending an Emergency Department

**DOI:** 10.1016/j.cjco.2026.02.015

**Published:** 2026-02-27

**Authors:** Frederic Balen, Stephanie Louisot, Venkatesh Thiruganasambandamoorthy, Xavier Dubucs, Sandrine Charpentier

**Affiliations:** a Emergency Department, Centre Hospitalier Universitaire de Toulouse, Toulouse, France; b CERPOP – EQUITY, INSERM, Toulouse, France; c Ottawa Hospital Research Institute, The Ottawa Hospital, Ottawa, Ontario, Canada; d Emergency Department, Centre Hospitalier de Cahors, Cahors, France; e Department of Emergency Medicine, and School of Epidemiology and Public Health, University of Ottawa, Ottawa, Ontario, Canada; f Université Toulouse III, Toulouse, France

**Keywords:** palpitations, emergency department, risk factors, arrhythmia, triage

## Abstract

**Background:**

Palpitations are a common reason for calling 911 or visiting the emergency department (ED). The objective of this study was to identify risk factors of a serious underlying cause of palpitations that can be evaluated without an electrocardiogram (ECG).

**Methods:**

This observational cohort study took place from January 1 to June 30, 2019 in an academic ED. Patients aged > 18 years who visited the ED for palpitations were included. The primary outcome was a final diagnosis of a serious underlying cause of palpitations at ED discharge. We used backward stepwise logistic regression to identify independent risk factors of a serious cause of palpitations.

**Results:**

A total fo 215 patients were analyzed, including 80 (37%) with a serious underlying cause of palpitations. Four independent risk factors were associated with significant arrhythmia: age ≥ 50 years (odds ratio [OR] = 7.6; 95% confidence interval [CI] = 3.5-16.4), history of heart disease (OR = 4.7; 95% CI = 2.3-9.6), syncope as an associated symptom (OR = 11; 95% CI = 1.4-84.1), and tachycardia ≥ 100 beats per minute (OR = 3.8; 95% CI = 1.8-8.1). A total of 54 patients (25%) had no risk factors. Absence of a risk factor had a sensitivity of 96% (95% CI = 89%-99%), and a specificity of 38% (95% CI = 30%-46%).

**Conclusions:**

We found 4 independent risk factors associated with a serious underlying cause of palpitations at ED triage.

## Résumé

**Contexte:**

Les palpitations sont un motif fréquent d’appel au 911 ou de visite à l’urgence. L’objectif de cette étude consistait à déterminer les facteurs de risque prédisposant à une cause sous-jacente grave de palpitations, évaluables sans électrocardiogramme (ECG).

**Méthodologie:**

Il s’agit d’une étude de cohorte observationnelle menée entre le 1^er^ janvier et le 30 juin 2019 à l’urgence d’un hôpital universitaire. Étaient inclus les patients âgés de plus de 18 ans se présentant à l’urgence pour des palpitations. Le critère d’évaluation principal était le diagnostic final d’une cause sous-jacente grave de palpitations au moment du congé. Nous avons utilisé une régression logistique par étapes pour identifier les facteurs de risque indépendants d’une cause grave de palpitations.

**Résultats:**

Deux cent quinze patients ont été analysés, dont 80 (37 %) présentant une cause sous-jacente grave de palpitations. Quatre facteurs de risque indépendants ont été associés à une arythmie significative : âge ≥ 50 ans (rapport de cotes [RC] = 7,6; intervalle de confiance [IC] à 95 % = 3,5 à 16,4), antécédents de maladie cardiaque (RC = 4,7; IC à 95 % = 2,3 à 9,6), syncope en tant que symptôme associé (RC = 11; IC à 95 % = 1,4 à 84,1) et tachycardie ≥ 100 bpm (RC = 3,8; IC à 95 % = 1,8 à 8,1). Cinquante-quatre patients (25 %) ne présentaient aucun facteur de risque. La sensibilité et la spécificité de l’absence de facteurs de risque ont été respectivement de 96 % (IC à 95 % = 89 à 99 %) et de 38 % (IC à 95 % = 30 à 46 %).

**Conclusions:**

Nous avons mis en évidence quatre facteurs de risque indépendants associés à une cause sous-jacente grave de palpitations lors du triage à l’urgence.

Palpitations are a symptom described by patients as “flip-flopping” or “rapid fluttering in the chest” or “pounding in the neck.”^1^ They are a common reason for visiting emergency departments (EDs), general practitioners (GPs), and cardiologists.^2^ Persistent palpitations may also be the reason for calling 911. Palpitations are the reason for > 1% of ED visits.^3^ Emergency physicians seem to perceive this symptom as common and benign,^4^ even though some diagnoses that cause palpitations can be life-threatening (or, at least need to be identified and treated). Palpitations may be caused by cardiac arrhythmias (and potentially an underlying structural heart disease) that need to be diagnosed or investigated during ED visits.^1^^,^^2^

Most previous studies of palpitations have included patients in non-acute care settings, such as GP or cardiology clinics.^5^ Very few studies have focused on patients presenting with palpitations in the ED. Clinical and electrocardiogram (ECG) findings are essential for determining a diagnosis and prognosis of patients presenting with palpitations,^1^^,^^2^ but those examinations are not available at the point of triage or during 911 call assessment.

The objective of this study was to identify risk factors for a serious underlying cause of palpitations that can be evaluated without an ECG.

## Methods

### Study design and setting

This observational cohort study was conducted between January 1 and June 30, 2019, in the ED of a university hospital. In 2019, our ED recorded 117,000 visits across its 2 campuses, including 41,000 at the campus where the study was conducted. All cardiology units of the university hospital are located on this campus.

### Participants

Patients aged 18 years and older who presented to the ED with palpitations as their chief complaint were consecutively included in this chart review study. Exclusion criteria were as follows: having missing medical records and leaving the ED without being treated. This noninterventional study was registered by our Hospital Data Officer Protector (Commission Nationale de l'Informatique et des Libertés number: 2206723v0).

### Variables and data source

The primary outcome was a final diagnosis of a serious underlying cause of palpitations at hospital discharge. Palpitations were considered to have a “serious underlying cause” if they required treatment with medication, devices, or other intervention (such as cardioversion) during an ED stay or during hospitalization following the ED visit. Patients’ primary outcome and variables of interest were collected by S.L. from patients’ medical files (using Orbis software, Agfa HealthCare, Bordeaux, France). The recorded variables of interest, obtainable via patient interview, included the following: demographics (age, sex); medical history of heart disease, including prior arrhythmias, ischemic or valvular heart disease, or congestive heart failure; cardiovascular risk factors (smoking, diabetes, hypertension, hypercholesterolemia, and family history of heart disease); regular use of antiarrhythmic medications (β-blockers, calcium-channel blockers, amiodarone, and digoxin); and symptoms associated with palpitations (chest pain, syncope or near-syncope, dyspnea, and vagal symptoms). Vital signs at triage—particularly heart rate and systolic blood pressure— also were collected as potential predictors. Troponin levels and hospital admission after the ED visit were recorded to describe the study population.

### Statistical analysis

Data were analyzed with STATA software (version 16, StataCorp, College Station, TX). Patients with a final diagnosis of a serious underlying cause of palpitations were compared to patients without a serious underlying cause, using appropriate bivariate tests. Any histories of heart disease—including arrhythmia, ischemic or valvular heart disease, and other chronic heart failure—were combined into a single “history of heart disease” category for simplification, given that each condition was associated with an increased risk, albeit to varying degrees. Continuous variables associated with a serious underlying cause of palpitations (age and heart rate) were dichotomized using a threshold chosen using the Liu method. In particular, the optimal cutoff for heart rate was identified as 96 beats per minute (bpm) and was rounded to 100 bpm for practical purposes. Potential predictors associated with risk of a serious underlying cause of palpitation with a *P* < 0.2 in bivariate analysis were considered for multivariate analysis. Then, we derived a parsimonious model using a backward stepwise logistic regression by keeping only the significant variables (Supplemental Table S1). Only the final model is presented, and risk factors are described with odds ratios ORs and 95% confidence intervals (Cis). The test characteristics (sensitivity, specificity, and positive and negative likelihood ratio) of the presence of no risk factors were also calculated, with their 95% CI, using the binomial method.

## Results

During the 6-month inclusion period, 224 patients were admitted in the ED for palpitations. After excluding 9 patients (missing data), 215 patients were analyzed, including 80 (37%) with a serious underlying cause of palpitations. This cause was atrial fibrillation or flutter (n = 45; 56%), atrioventricular reentry tachycardia (n = 22; 27%), ventricular tachycardia (n = 5; 6%) or sinus tachycardia in specific context (n = 8; 10%; ie, acute heart failure [n = 3], pulmonary embolism [n = 2] or acute severe hypertension [n = 3]). Patients’ characteristics are shown in Table 1.

**Table 1:** Patients' characteristics

Characteristic	Population	No serious underlying cause	Serious underlying cause	*P*
	(n = 215)	(n = 135)	(n = 80)	
Age, Y	49 ± 19	42 ± 17	62 ± 17	< 0.001
age ≥ 50 Y	104 (48)	42 (31)	62 (78)	< 0.001
Women	113 (52)	69 (51)	44 (55)	0.581
History of cardiac disease	84 (39)	31 (23)	53 (66)	< 0.001
Arrhythmia	68 (32)	21 (16)	47 (59)	< 0.001
Ischemic cardiopathy	13 (6)	5 (4)	8 (10)	0.077
Valvular cardiopathy	11 (5)	5 (4)	6 (8)	0.337
Other	5 (2)	3 (2)	2 (3)	1
Cardiovascular usual risk factors				
Smoker	65 (30)	46 (34)	19 (24)	0.111
Diabetes	18 (8)	8 (6)	10 (13)	0.093
Hypertension	60 (28)	24 (18)	36 (45)	< 0.001
Hypercholesterolemia	28 (13)	15 (11)	13 (16)	0.279
Family history of heart disease	36 (17)	21 (16)	15 (19)	0.544
Usual treatment				
β-blockers	49 (23)	20 (15)	29 (36)	< 0.001
Calcium-channel blocker	6 (3)	2 (1)	4 (5)	0.198
Amiodarone	5 (2)	1 (1)	4 (5)	0.065
Digoxin	9 (4)	1 (1)	8 (10)	0.002
Associated symptoms:				
Chest pain	82 (38)	58 (43)	24 (30)	0.059
Syncope	6 (3)	2 (1)	4 (5)	0.198
Near syncope	23 (11)	13 (10)	10 (13)	0.51
Dyspnea	34 (16)	20 (15)	14 (18)	0.602
Vagal symptoms	37 (17)	26 (19)	11 (14)	0.301
Vital parameters at triage				
HR, bpm	90 (78–107)	88 (78–101)	98 (81–135)	0.003
SBP, mm Hg	133 (121–148)	133 (122–148)	134 (120–147)	0.845
Blood test				
Initial Troponin∗	< 7 (<7–15)	< 7 (<7–8)	12 (<7–47)	N.A.
Hospital admission after ED	18 (8)	4 (3)	14 (18)	N.A.

Values are mean ± standard deviation, n (%), or median (interquartile range), unless otherwise indicated.

bpm, beats per minute; ED, emergency department; HR, heart rate; N.A., not applicable; SBP, systolic blood pressure;.

∗ Missing values: 96 (45%)—56 (41%) in no significant–arrhythmia group and 40 (50%) in significant-arrhythmia group.

Four independent risk factors were associated with a serious underlying cause of palpitations: age ≥ 50 years (OR = 7.6; 95% CI = 3.5-16.4), history of heart disease (OR = 4.7; 95% CI = 2.3-9.6), syncope as an associated symptom (OR = 11; 95% CI = 1.4-84.1), and tachycardia ≥ 100 bpm (OR = 3.8; 95% CI = 1.8-8.1; Table 2).

**Table 2:** Risk factors of serious underlying cause of palpitations (area under the curve = 0.839)

Characteristic	OR [95% CI]		*β coef* [95% CI]		*P*
Age ≥ 50 Y	7.6	[3.5–16.4]	2	[1.3–2.8]	< 0.001
History of heart disease	4.7	[2.3–9.6]	1.6	[0.8–2.3]	< 0.001
Syncope	11.0	[1.4–84.1]	2.4	[0.36–4.4]	0.021
Heart rate ≥ 100 bpm	3.8	[1.8–8.1]	1.3	[0.6–2.1]	< 0.001

bpm, beats per minute; CI, confidence interval; coef, coefficient; OR, odds ratio.

A total of 54 patients (25%) had no risk factors, including 3 patients (6%) presenting with a serious underlying cause of palpitations (such as atrial fibrillation [n = 2] and atrioventricular reentry tachycardia [n = 1]). Absence of a risk factor had a sensitivity of 96% (95% CI = 89%-99%) and a specificity of 38% (95% CI = 30%-46%) (Table 3).

**Table 3:** Performance of risk factor detection sensibility is an inappropriate translation of sensitivity indeed

Risk factor presence	Serious underlying cause of palpitations	No serious underlying cause of palpitations	Total	
Risk factor (≥ 1)	77	84	161	LR + = 1.55 (95% CI = [1.35–1.78])
No risk factor	3	51	54	LR - = 0.01 (95% CI = [0.03–0.31])
Total	80	135	215	
	Se = 96%	Sp = 38%		
	(95% CI = [89–99])	(95% CI = [30–46])		

CI, confidence interval; LR +/-, positive and negative likelihood ratio; Se, sensibility; Sp, specificity.

## Discussion

### Interpretation of findings

We identified 4 independent risk factors associated with a serious underlying cause of palpitations: age ≥ 50 years, history of heart disease, syncope as an associated symptom, and tachycardia (≥ 100 bpm). These risk factors can be assessed easily during triage or the initial clinical evaluation. Although our model cannot safely rule out a serious underlying cause without further investigation, it may assist 911 dispatchers and triage nurses in prioritizing patients, and support physicians in their clinical decision-making.

### Comparison to previous studies

We report a 37% prevalence of a serious underlying cause of palpitations, consistent with the largest ED-based study on palpitations, in which a 34% prevalence of cardiac causes was observed.^3^ Older age has already been described in the primary care setting to be associated with significant arrhythmia.^6^ History of heart disease was also reported to be associated with cardiac arrhythmia.^7^ Patients admitted in ED for syncope and palpitations are likely to be registered with “syncope” as their chief complaint. This may explain why, in “palpitation-centered” cohorts, syncope or presyncope was not identified as a risk factor.^5^ However, the association between syncope and palpitations with arrhythmia is not surprising, given that syncope may result from severe arrhythmias. High heart rate already has been described to be associated with untreated atrial tachycardia or flutter.^2^

### Strengths and limitations

First, due to the retrospective design, other symptoms reported in the literature for patients consulting a GP or cardiologist were not evaluated, as these questions are not routinely asked during ED visits (eg, duration or persistence of palpitations, or whether palpitations occur during sleep or work).^5^ Second, the small number of patients presenting with syncope led to wide 95% CIs, highlighting potential model instability and raising concerns about overfitting. Third, the limited sample size did not allow for internal validation of the proposed model. Nevertheless, to our knowledge, this study is the first on this topic in an ED setting. Fourth, the occurrence of a serious underlying cause of palpitations after hospital discharge was not assessed; however, we hypothesize that such events are rare following an appropriate ED evaluation. Moreover, our study was designed to provide a prioritization aid rather than a rule-out strategy. Finally, patients were recruited during the ED visit rather than in the prehospital setting, and the validity of our findings in this context remains to be determined.

### Potential clinical implications

Our model has insufficient sensitivity to safely rule out a serious underlying cause of palpitations without an ECG. However, in prehospital settings, it may assist dispatchers and paramedics in decision-making by stratifying the risk of a serious underlying cause. The identified risk factors can be assessed using patient history and heart rate measurements. Heart rate can be self-monitored—potentially with a smartwatch or smartphone application—or measured by paramedics. Patients without risk factors likely could be referred to a GP, provided one is available and is capable of performing an ECG. Otherwise, these patients may require less-urgent ambulance dispatch and could be transported to specialized noncardiology centres. At ED triage, our model may guide pre-ECG prioritization but should not be used alone to rule out the need for an ECG during an ED visit. Moreover, patients with risk factors may require further evaluation for cardiac arrhythmia after a single ECG, such as blood tests, echocardiography, or Holter monitoring. After ED discharge, new devices may help to diagnose cardiac arrhythmia in patients without significant findings during an ED visit.^8^

### Research implications

A prospective cohort study is needed to validate these findings, especially in prehospital settings. Considering initial ECG findings as potential predictors may prove interesting. Moreover, future studies should ensure the absence of post-discharge events, regardless of the proposed model.

## Conclusion

One third of patients presenting at the ED for palpitations have a serious underlying cause. We found 4 independent risk factors associated with a serious underlying cause of palpitations that can be assessed at ED triage: age ≥ 50 years, history of heart disease, syncope as an associated symptom, and tachycardia ≥ 100 bpm.

## Ethics Statement

This retrospective observational study was reported in compliance with our institution’s requirements. The research reported in this paper adhered to STROBE guidelines.

## Patient Consent

The authors confirm that patient consent is not applicable to this article. This is a retrospective cohort study using de-identified data; therefore the IRB did not require consent from patient. However, patients are informed that their data may be used for research purposes when visiting our hospital.

## Funding Sources

The authors have no funding sources to declare.

## Disclosures

The authors have no conflicts of interest to disclose.

## Footnotes

To access the supplementary material accompanying this article, visit *CJC Open* at https://www.cjcopen.ca/ and at https://doi.org/10.1016/j.cjco.2026.02.015
