# Supplementary material for: Risk Factors of a Serious Underlying Cause of Palpitations Among Patients Attending an Emergency Department
Source: CJC Open. 2026 Feb 27;8(7):796–9. doi: 10.1016/j.cjco.2026.02.015 (PMC13386736; doi:10.1016/j.cjco.2026.02.015)
Supplement: Supplementary File [file mmc1.pdf]

Supplemental Table S1. Multivariate analyses

	Full Model OR [95%CI]		Heart disease not grouped OR [95%CI]		Final Model OR [95%CI]	
Age ≥ 55 years	7.0	[2.9 - 16.7]	8.1	[3.7 - 17.8]	7.6	[3.5 - 16.4]
History of heart disease:						
- Arrythmia	7.2	[2.9 - 17.8]	6.8	[3.2 - 14.4]		
- Ischaemic cardiopathy	2.6	[0.5 - 12.8]	2.2	[0.5 - 9.7]	4.7	[2.3 - 9.6]
- Valvular cardiopathy	1.6	[0.3 - 8.0]	1.5	[0.3 - 7.1]		
- Other	omitted		omitted			
Cardiovascular usual risk factors:						
- Smoker	0.6	[0.3 - 1.4]				
- Diabetes	1.2	[0.3 - 4.0]				
- Hypertension	1.3	[0.5 - 3.4]				
Usual treatment:						
- βblockers	0.8	[0.3 - 3.4]				
- Calcium channel blocker	0.7	[0.1 - 6.6]				
- Amiodarone	0.8	[0.1 - 10.3]				
- Digoxin	3.9	[0.4 - 38.1]				
Associated symptoms:						
- Chest pain	0.6	[0.3 - 1.2]				
- Syncope	13.2	[1.5 - 112.1]	11.5	[1.4 - 92.6]	11.0	[1.4 - 84.1]
Heart rate ≥ 100 bpm	3.8	[1.7 - 8.4]	3.7	[1.7 - 8.0]	3.8	[1.8 - 8.1]
AUC	0.863		0.851		0.839	
